# Gut microbiome–metabolome signatures of osteosarcopenia in fracture patients in China

**DOI:** 10.3389/fendo.2026.1863988

**Published:** 2026-09-04

**Authors:** Minjuan Li, Xian Zhao, Bin Zhang, Renwei Cao, Zhongyu Wang, Zishuai Huang, Cheng Cheng, Shuai Lu, Xieyuan Jiang

**Affiliations:** 1 Department of Orthopedic Trauma, Beijing Jishuitan Hospital, Capital Medical University, Beijing, China; 2 Beijing Research Institute of Traumatology and Orthopaedics, Beijing, China; 3 Laboratory for Clinical Medicine, Capital Medical University, Beijing, China; 4 Department of Osteoporosis, Beijing Jishuitan Hospital, Capital Medical University, Beijing, China

**Keywords:** gut microbiome, lipid mediators, osteosarcopenia, short-chain fatty acids, shotgun metagenomics, untargeted metabolomics

## Abstract

**Background:**

Osteosarcopenia, defined as the coexistence of low bone mass and sarcopenia, is a disabling musculoskeletal condition, yet its gut microbial and metabolic characteristics in clinical populations remain incompletely understood. Integrative multi-omics approaches may help clarify species–metabolite networks associated with this condition, particularly in fracture patients.

**Methods:**

In this single-center, prospective cross-sectional study, 69 fracture patients aged ≥50 years were classified into four phenotypes: Normal (n = 18), isolated low bone mass (Bone, n = 18), isolated sarcopenia (Muscle, n = 19), and osteosarcopenia (Both, n = 14). Fecal samples were analyzed using shotgun metagenomics and untargeted metabolomics, yielding paired multi-omics data for 52 participants.

**Results:**

The Bone group had the highest mean age (66.6 ± 9.46 years), whereas the mean ages of the other groups ranged from 60.6 to 61.8 years (overall *p* = 0.029), while sex, BMI, lifestyle factors, and comorbidities did not differ significantly. Neither α-diversity nor overall β-diversity showed marked differences across phenotypes, suggesting that broad community replacement was not observed. A multi-stage, multi-method strategy yielded a 17-species consensus feature set associated with differences among musculoskeletal phenotypes. Taxonomic patterns were consistent with a candidate fiber/short-chain fatty acid (SCFA)-associated module, whereas exploratory microbe–metabolite correlations suggested a candidate lipid/sterol-associated module. The latter included correlations linking *Firmicutes bacterium* CAG:24053_14 with putatively annotated cholesterol and N-acylethanolamines.

**Conclusions:**

Osteosarcopenia in fracture patients was associated with unadjusted differences in selected gut microbial taxa and fecal metabolites within a broadly shared microbial community. These findings are hypothesis-generating and require validation in larger independent cohorts before clinical or biomarker application.

## 1 Introduction

Population aging has placed age-related musculoskeletal disorders at the center of public health. Sarcopenia and low bone mass (osteopenia or osteoporosis) are common in older adults, increasing the risks of falls and fractures and accelerating functional decline (1, 2). Sarcopenia is a progressive and generalized disorder defined primarily by reduced muscle strength, with concomitant losses in muscle mass or quality and physical performance, whereas osteoporosis is characterized by low bone mineral density (BMD) with microarchitectural deterioration of bone tissue (1, 2). In patients with fractures, these disorders frequently coexist and are associated with poorer clinical outcomes (3). The muscle–bone unit exemplifies the close interdependence of these tissues across anatomic, mechanical, vascular, and endocrine levels. Beyond mechanical loading, myokines (e.g., myostatin, irisin) and osteokines (e.g., osteocalcin, sclerostin) mediate bidirectional crosstalk via shared signaling pathways (4). This biological interconnection provides a pathophysiologic basis for the concept of osteosarcopenia—the coexistence of sarcopenia and low bone mass, which is now widely recognized as a distinct musculoskeletal phenotype associated with increased risks of falls, fractures, disability, and mortality (5–7).

Over the past decade, the gut microbiota has emerged as a key environmental determinant and a virtual endocrine organ, shaping host metabolism, immunity, mucosal integrity, and neuroendocrine homeostasis (8–10). In postmenopausal osteoporosis, recent analyses have linked microbial diversity and specific taxa to bone turnover markers and metabolite profiles (10). In older adults with low muscle mass, reduced fecal butyrate and distinct microbial signatures indicate disrupted gut–muscle metabolic interactions (11–13). Mechanistically, converging data highlight interconnected gut–muscle and gut–bone axes. Microbial dysbiosis promotes inflammaging and endotoxemia, with lipopolysaccharide (LPS) signaling impairing muscle protein synthesis and stimulating osteoclast-mediated bone resorption. Conversely, microbially derived short-chain fatty acids (SCFAs)—notably butyrate—enhance osteoblastogenesis, inhibit osteoclast differentiation, and support muscle anabolism (14, 15). With advancing age, compositional and functional drifts of the microbiome may destabilize this musculoskeletal homeostasis, predisposing to bone and muscle loss (16).

Among patients with fractures, differences in gut microbial composition and metabolism across musculoskeletal phenotypes may represent important environmental determinants and more closely reflect pre-fracture predisposition than immediate post-injury changes (17, 18). Many prior studies of osteoporosis or sarcopenia have relied on 16S rRNA gene sequencing, whose resolution is typically limited to the genus level and provides only indirect functional inference (19, 20). By contrast, shotgun metagenomics enables species-level resolution and direct functional gene profiling to interrogate functional and metabolic pathways. When integrated with untargeted metabolomics, this multi-omics framework can align microbial features with fecal metabolite profiles and generate cross-domain hypotheses, although such associations do not establish causal or mechanistic relationships (11, 21, 22). We therefore hypothesized that fracture patients with different musculoskeletal phenotypes would exhibit distinguishable gut microbial and metabolite profiles.

Accordingly, this study aimed to characterize cross-sectional gut microbial and metabolite differences among fracture patients with four musculoskeletal phenotypes: Normal, isolated low bone mass (Bone), operationally defined isolated sarcopenia (Muscle), and the coexistence of low bone mass and operationally defined sarcopenia (Both). By integrating shotgun metagenomics with untargeted metabolomics, we further sought to identify candidate taxa–metabolite associations and predicted functional modules. Given the exploratory and cross-sectional design, these analyses were intended to generate hypotheses for future validation rather than establish causal mechanisms, diagnostic biomarkers, or therapeutic targets.

## 2 Materials and methods

### 2.1 Study design and ethics

This single-center, prospective cross-sectional study was conducted at Beijing Jishuitan Hospital in accordance with the Declaration of Helsinki. The study protocol was approved by the institutional ethics committee (Approval No. K2024-273). Written informed consent was obtained from all participants prior to enrollment.

### 2.2 Participants and recruitment

Consecutive patients with fractures were recruited from the Department of Orthopedic Trauma between May 2024 and September 2024.

- Inclusion criteria. (i) Age ≥ 50 years; (ii) fracture confirmed by imaging; (iii) hemodynamic stability; (iv) admission ≤ 72 h after injury.
- Exclusion criteria. (i) Pathological fractures due to malignancy, infection, or other non-osteoporotic bone diseases; (ii) systemic antibiotics or probiotics within the preceding 30 days; (iii) oral or parenteral glucocorticoids for > 3 months cumulatively within the past 6 months; (iv) disorders with major effects on bone metabolism (e.g., hyperthyroidism, end-stage renal disease, Paget’s disease); (v) inflammatory bowel disease; (vi) history of major gastrointestinal surgery; (vii) acute gastrointestinal infection; (viii) hepatic failure; (ix) autoimmune disease; (x) bowel cleansing/colonoscopy within the previous 2 weeks; (xi) extreme diets.

To minimize iatrogenic perturbations after fracture, all fecal samples were collected immediately upon admission and strictly before any microbiota-modifying procedure (e.g., surgery, perioperative antibiotics, bowel preparation).

### 2.3 Diagnostic criteria and grouping

Participants were classified into four musculoskeletal phenotypes:

- Normal: normal bone and muscle status;
- Bone: isolated low bone mass (osteopenia or osteoporosis) without sarcopenia;
- Muscle: isolated sarcopenia without low bone mass;
- Both: osteosarcopenia.

An Abnormal composite group comprising the Bone, Muscle and Both phenotypes was defined for primary analyses.

Bone status followed WHO criteria. Bone mineral density (BMD) was measured by dual-energy X-ray absorptiometry (DXA) at the lumbar spine (L1–L4), femoral neck, or total hip. Participants were categorized as follows: Normal: T-score ≥ −1.0; Osteopenia: −2.5 < T-score < −1.0; Osteoporosis: T-score ≤ −2.5 (2). Sarcopenia was defined by EWGSOP2 with Asian thresholds. Muscle status was operationally classified using the SARC-F, handgrip strength, and calf circumference based on Asian thresholds. Screening was performed using the SARC-F (score ≥4), followed by assessment of handgrip strength (<28 kg for men or <18 kg for women), using the best of three trials on the dominant hand. Because appendicular skeletal muscle mass was not measured using DXA or BIA, low muscle mass was approximated using calf circumference (<34 cm for men and <33 cm for women). Accordingly, the Muscle and Both groups should be interpreted as operationally defined sarcopenia phenotypes rather than imaging-confirmed sarcopenia (23).

### 2.4 Clinical data collection

Demographics and anthropometric data (age, sex, height, weight, body mass index [BMI]), comorbidities (e.g., diabetes, cardiovascular disease), medication use (e.g., glucocorticoids, proton-pump inhibitors), lifestyle factors (smoking, alcohol, tea consumption), regular physical activity, and laboratory parameters were recorded.

### 2.5 Sample collection and storage

Fresh fecal samples were collected, homogenized, and divided into two equal aliquots: one for shotgun metagenomic sequencing and the other for untargeted metabolomic analysis. All samples were frozen at −80 °C. A pooled quality-control (QC) sample representing all participants was prepared for metabolomics analysis.

### 2.6 Shotgun metagenomic sequencing

#### 2.6.1 Metagenomic data processing and annotation

Microbial DNA was extracted using the Fecal Genome DNA Extraction Kit (BioTeke, AU46111-96, China). Unused swabs processed in parallel served as blank controls. DNA was quantified with the Qubit 1X dsDNA HS Assay Kit (Invitrogen, Q33230), eluted in 50 µL buffer, and stored at −80 °C. Quantification, library preparation, and sequencing were performed by LC-Bio Technology (Hangzhou, China). Paired-end DNA libraries were prepared using the TruSeq Nano DNA LT kit (Illumina, FC-121-4001). Approximately 200 ng DNA per sample was fragmented to 200–500 bp with a Bioruptor Pico (Diagenode), followed by end repair, A-tailing, adapter ligation, and PCR amplification (8 cycles). Libraries were sequenced on an Illumina NovaSeq 6000 platform (paired-end 150 bp).

Raw reads were quality-filtered using fastp (v0.23.4; parameters: −l 100, −g, −W 6, −5, −q 20, −u 30). High-quality reads were mapped to the human genome (GRCh38/hg38) with Bowtie2 (v2.2.0) to remove host sequences. Remaining non-host reads were *de novo* assembled per sample using MEGAHIT (v1.2.9), retaining contigs ≥ 500 bp.

Coding sequences (CDSs) were predicted with MetaGeneMark (v3.26), and predicted CDSs <100 nt were discarded. All predicted genes were clustered with MMseqs2 (v15-6f452; identity ≥95%, coverage ≥90%) to build a non-redundant unigene catalog. Clean non-host reads were mapped back to the unigene catalog with Bowtie2; unigenes with ≤2 total mapped reads across all samples were excluded. Taxonomic annotation of unigene protein sequences was performed using DIAMOND (v0.9.14, blastp) against the NR_meta database at the species level. Functional annotation was conducted using the KEGG database.

#### 2.6.2 Community diversity and differential abundance analyses

α-diversity (Observed species, Shannon) was computed using Quantitative Insights Into Microbial Ecology, version 1 (QIIME1). β-diversity was calculated using Bray–Curtis dissimilarity and visualized by Principal Coordinates Analysis (PCoA). Group differences in community structure were assessed using PERMANOVA (adonis2, vegan R package), with homogeneity of dispersions assessed by betadisper.

Differentially abundant taxa between groups were identified using multiple complementary approaches. The Wilcoxon rank-sum test screened for species-level differences (top 20 by rank, *p* < 0.05). LEfSe (linear discriminant analysis effect size) analysis was applied to detect discriminant taxa. A binary random forest classifier (Normal vs. Abnormal) was trained in scikit-learn with stratified 5-fold cross-validation. Feature importance was ranked using the permutation-based mean decrease in accuracy (MDA), and the top 10 taxa were reported. Two-group comparisons were further evaluated using STAMP v2.1.3.

To enhance robustness and minimize method-specific bias, consensus feature taxa were defined as species supported by at least two analytical approaches. In the primary Normal-versus-Abnormal comparison, four complementary methods were applied: Wilcoxon testing, LEfSe, random forest, and STAMP. Secondary cross-sectional comparisons were performed among the Normal, Bone, and Both groups and among the Normal, Muscle, and Both groups using nonparametric testing and LEfSe. Species supported by at least two analytical approaches across the corresponding comparison framework were retained. After pooling eligible taxa from the primary and secondary comparisons and removing duplicates, a final 17-species consensus feature set was generated for downstream descriptive and microbe–metabolite analyses.

### 2.7 Untargeted fecal metabolomics

Approximately 20 mg feces were homogenized with 1 mL ice-cold 50% methanol, vortexed, incubated at room temperature for 10 min, and precipitated at −20 °C overnight. The next day, samples were centrifuged at 4 °C (4,000×g, 20 min), and supernatants were transferred to 96-well plates. Extracts were stored at −80 °C until LC–MS analysis. Chromatographic separation was performed on a Vanquish Flex UHPLC system (Thermo Fisher Scientific, USA) with an ACQUITY UPLC BEH C18 column (1.8 µm, 2.1 mm × 100 mm; Waters, USA) or an equivalent reversed-phase column. Mobile phases were 0.1% formic acid in water (A) and acetonitrile (B). Gradient: 0–0.5 min, 5% B; 0.5–7.0 min, linear to 100% B; 7.0–8.0 min, 100% B; 8.0–8.1 min, 100%→5% B; 8.1–10.0 min, 5% B. Data were acquired on an Orbitrap Exploris 480 in positive and negative ion modes (MS1 m/z 70–1050, resolution 70,000; DDA-MS2 Top 3 with HCD, resolution 17,500).

Raw files (.raw) were converted to mzXML and processed in R using XCMS (nonlinear retention-time alignment, peak detection, and feature integration) and CAMERA (annotation of isotopes, adducts, and in-source fragments). Metabolites were putatively annotated using accurate mass (<10 ppm) with database matching against KEGG. For features with available MS/MS spectra, fragmentation patterns were additionally compared with an in-house spectral library. Because authentic chemical standards were not analyzed under identical experimental conditions, none of the named metabolites met MSI level 1 identification. Key lipid-related metabolites supported by MS/MS matching were classified as MSI level 2 (putatively annotated compounds) and were not regarded as definitively identified (24). The feature matrix was preprocessed in metaX: (1) exclude features missing in >50% of QC or >80% of biological samples; (2) impute remaining missing values using k-nearest neighbors; (3) remove features with QC RSD >30%.

### 2.8 Multi-omics integration

Spearman’s rank correlation was used to explore associations between the relative abundances of the 17 consensus feature species and the normalized intensities of differential fecal metabolites. Correlation pairs meeting |ρ| ≥0.7 and nominal p<0.05 were retained for exploratory network construction. The relatively stringent correlation threshold was used to prioritize strong associations and improve network specificity and interpretability, while accepting that weaker associations might be excluded. No multiple-testing correction was applied to these correlations. Accordingly, the resulting network was considered hypothesis-generating, and individual edges were not interpreted as confirmatory evidence of direct microbial–metabolite interactions.

### 2.9 Statistical analysis and graphics

All analyses were performed in R v4.5.0. Data normality was assessed using the Shapiro–Wilk test. Two-group comparisons were performed using Student’s t-test or Mann–Whitney U test, as appropriate. For comparisons involving three or more groups, one-way ANOVA with Tukey’s HSD or Kruskal–Wallis with Dunn’s *post hoc* test was applied. Categorical variables were compared using the χ² test or Fisher’s exact test. Unless otherwise specified, *p* < 0.05 was considered statistically significant.

Data visualization included Venn diagrams, Sankey plots, chord diagrams, genus-level Manhattan plots for screening (−log10 P), hierarchical heatmaps (candidate taxa), and correlation network visualizations based on significant Spearman associations (microbe–metabolite edges). All plots were generated in R using appropriate packages. Microbiome differential-abundance and microbe–metabolite correlation analyses were not adjusted for age. Therefore, the observed phenotype-associated findings, particularly those involving the older Bone group, cannot be interpreted as independent of age.

## 3 Results

### 3.1 Demographic and clinical characteristics of participants

**Figure 1:**
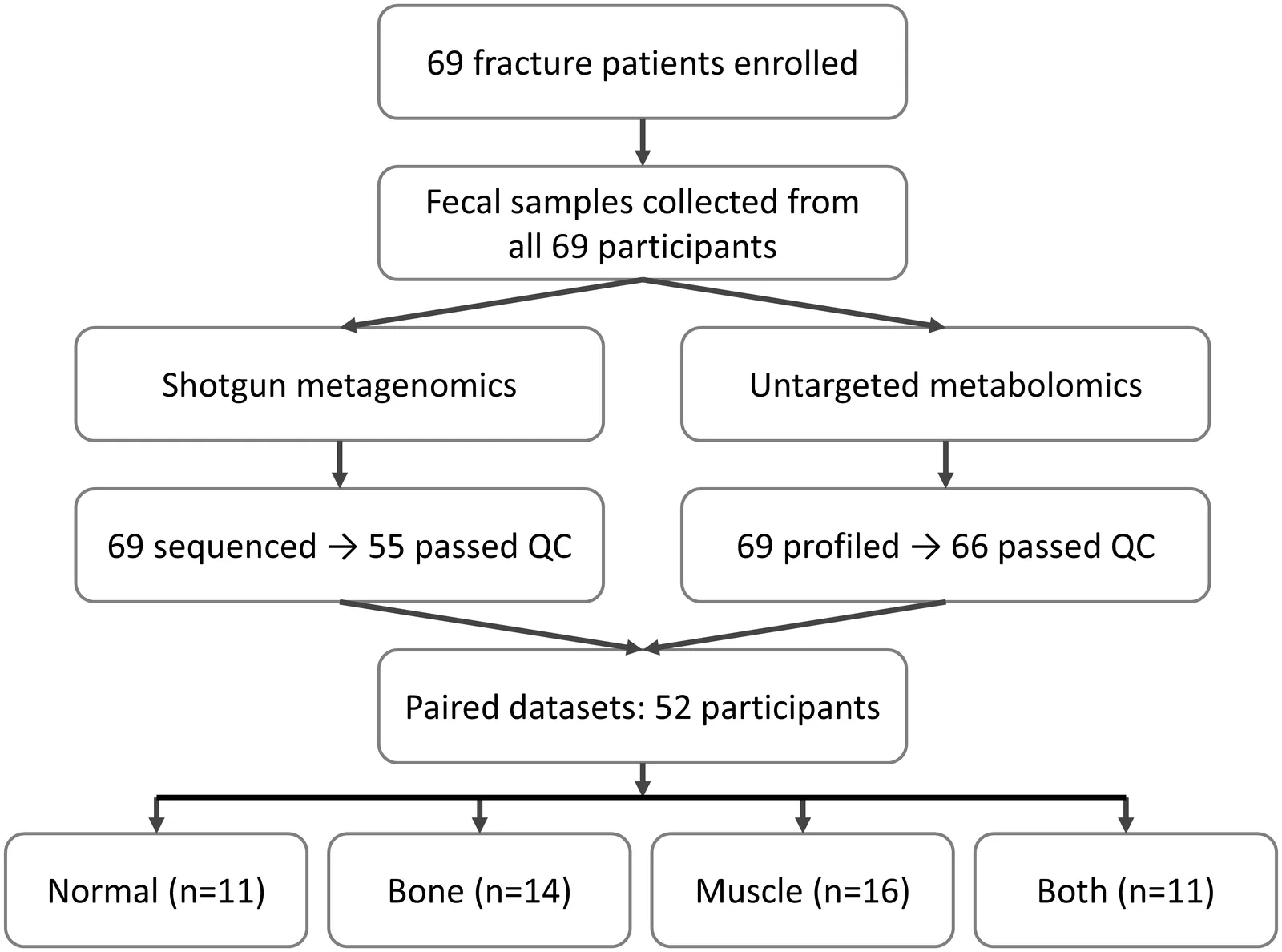
Study flow diagram and allocation of paired multi-omics datasets.

We enrolled 69 patients with fractures, who were classified into four musculoskeletal phenotypes: Normal (n=18), Bone (n=18), Muscle (n=19), and Both (n=14). All 69 fecal samples underwent both shotgun metagenomic sequencing and untargeted metabolomic profiling; 55 metagenomes and 66 metabolomes passed quality control. Paired datasets for both assays were available for 52 participants (Normal, n=11; Bone, n=14; Muscle, n=16; Both, n=11). Participant flow and omics data allocation are illustrated in **Figure 1**.

Age differed significantly across groups (*p* = 0.029), with the Bone group showing the highest mean age (66.6 ± 9.46 years), while the other groups ranged from 60.6 to 61.8 years. No statistically significant between-group differences were observed in sex distribution, BMI, lifestyle factors (smoking, alcohol, tea consumption), prevalence of cardiovascular diseases or metabolic disorders, or regular physical activity (all *p* > 0.05). The overall distribution of assay coverage (dual-omics vs single-omics samples) did not differ significantly among groups (*p* = 0.555). The measured demographic and clinical characteristics of participants with paired metagenomic–metabolomic data (n = 52) were comparable to those of the full cohort, although selection bias related to unmeasured factors cannot be excluded. A summary of demographic and clinical characteristics is provided in **Table 1**.

**Table 1:** Demographic and clinical characteristics among the patient groups.

Characteristic	Normal (n=18)	Low bone mass (n=18)	Sarcopenia (n=19)	Osteosarcopenia (n=14)	*P*
Age, years	60.8 ± 5.04	66.6 ± 9.46	60.6 ± 4.93	61.8 ± 6.32	0.029
Sex (Female), n (%)	11(61.1)	11(61.1)	10(52.6)	11(78.6)	0.458
BMI, kg/m^2^	25.4 ± 3.19	24.6 ± 2.61	25.6 ± 2.90	23.8 ± 2.52	0.274
Smoking, n (%)	2(11.1)	4(22.2)	4(21.1)	3(21.4)	0.758
Alcohol drinking, n (%)	3(16.7)	3(16.7)	4(21.1)	3(21.4)	0.969
Tea drinking, n (%)	3(16.7)	2(11.1)	3(15.8)	5(35.7)	0.320
Cardiovascular diseases, n (%)	8(44.4)	9(50.0)	10(52.6)	8(57.1)	0.846
Metabolic disorders, n (%)	4(22.2)	6(33.3)	4(21.1)	3(21.4)	0.712
Physical Exercise, n (%)	3(16.7)	2(11.1)	4(21.1)	3(21.4)	0.846
Assay participation					0.555
-Both Metagenomics and Metabolomics, n (%)	11(61.1)	14(77.8)	16(84.2)	11(78.6)	
-Only Metagenomics, n (%)	2(11.1)	1(5.6)	0(0.0)	0(0.0)	
-Only Metabolomics, n (%)	5(27.8)	3(16.7)	3(15.8)	3(21.4)	

BMI, body mass index.

Cardiovascular diseases include hypertension and coronary heart disease. Metabolic disorders include diabetes mellitus and hyperlipidemia.

### 3.2 Community diversity and differential taxa between normal and abnormal groups

**Figure 2:**
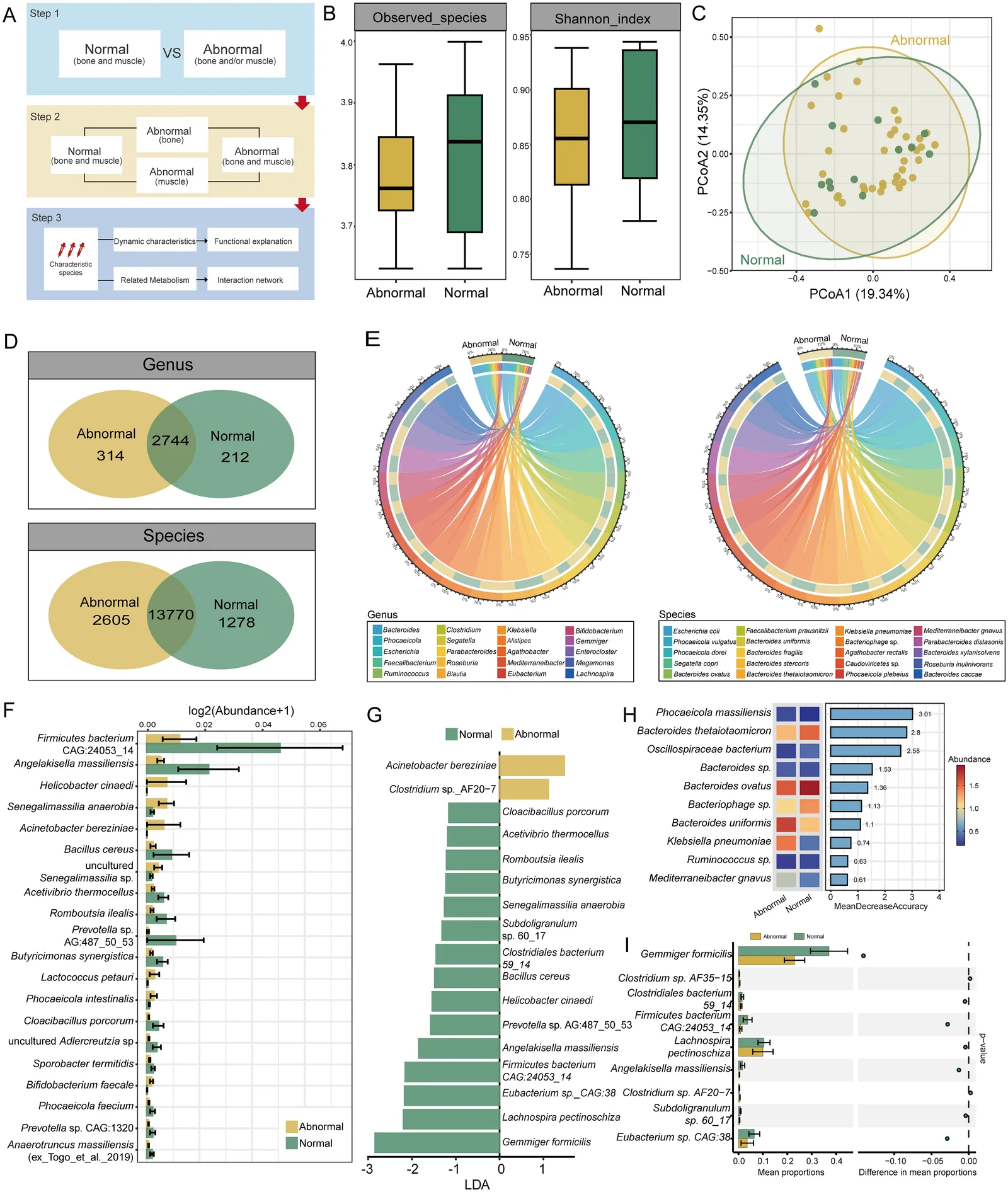
**(a)** Overview of the analytical workflow. Step 1: participants were classified into Normal and Abnormal group. Step 2: the Abnormal group was further stratified into Bone (isolated low bone mass), Muscle (isolated sarcopenia), and Both (osteosarcopenia). Secondary cross-sectional comparisons were conducted among the Normal, Bone, and Both groups and among the Normal, Muscle, and Both groups. Step 3: differential taxa were screened and subjected to descriptive and predicted functional interpretation and microbe–metabolite network analyses. **(b)** α-diversity (Observed species and Shannon index) in Normal (green) versus Abnormal (yellow). Both indices were numerically lower in the Abnormal group, but neither difference reached statistical significance. **(c)** β-diversity PCoA (Bray–Curtis) showing overlapping community structures between Normal and Abnormal (PCoA1 = 19.34%, PCoA2 = 14.35%). **(d)** Venn diagrams showing shared and unique taxa between groups at genus and species levels. **(e)** Chord diagrams illustrating the distribution and sharing of the top 20 genera and top 20 species by mean relative abundance. **(f)** Species showing nominal between-group differences in the Wilcoxon rank-sum test (*p* < 0.05); top 20 by mean relative abundance are shown (log_2_[Abundance + 1]). **(g)** LEfSe analysis (LDA > 2, *p* < 0.05) identifying four Normal-enriched species and no Abnormal-enriched species. **(h)** Random forest classifier distinguishing Normal and Abnormal, with the top 10 species ranked by MeanDecreaseAccuracy and corresponding heatmap of mean relative abundances. **(i)** STAMP analysis identifying nine species with nominal *p* < 0.05 based on group mean differences and corresponding significance.

We first compared the fecal microbiome between the Normal and Abnormal groups. An overview of the analytical workflow is shown in **Figure 2a**. At the species level, the α-diversity indices (Observed species and Shannon) were numerically lower in the Abnormal group than in the Normal group, although neither difference was statistically significant (**Figure 2b**). Similarly, β-diversity by PCoA revealed overlapping centroids without a distinct separation between the two groups (**Figure 2c**). Taxonomic breadth analyses indicated a large shared core alongside group-specific constituents (**Figure 2d**). Chord diagrams of the top 20 genera and species further illustrated broad overlap with shifts in the relative contribution of selected taxa (**Figure 2e**).

At the differential-abundance level, the Wilcoxon test identified 1,057 species showing nominal between-group differences (*p* < 0.05); the top 20 ranked by mean abundance are displayed (**Figure 2f**). These nominal results were used for exploratory feature screening rather than interpreted as confirmatory differential-abundance findings. Complementary LEfSe analysis (LDA > 2, *p* < 0.05) detected no Abnormal-enriched species, whereas four species were enriched in the Normal group—*Firmicutes bacterium* CAG:24053_14, *Eubacterium* sp. *CAG:38*, *Lachnospira pectinoschiza*, and *Gemmiger formicilis* (**Figure 2g**). A random forest classifier highlighted a partially overlapping set of high-importance species, summarized by Mean Decrease Accuracy (**Figure 2h**). Using STAMP, nine species showed between-group differences at nominal p < 0.05 (**Figure 2i**).

To enhance robustness and reduce method-specific bias, we defined “consensus feature taxa” as species repeatedly flagged by ≥2 approaches (Wilcoxon, LEfSe, random forest, STAMP). This cross-method criterion yielded five species—*Angelakisella massiliensis*, *Eubacterium* sp. CAG:38, *Firmicutes bacterium* CAG:24053_14, *Gemmiger formicilis*, and *Lachnospira pectinoschiza*. These five species constituted the initial cross-method consensus set and were carried forward into the secondary cross-sectional comparisons among the Normal, Bone, and Both groups and among the Normal, Muscle, and Both groups.

### 3.3 Cross-sectional microbiome profiles across the normal, bone, muscle, and both phenotypes

**Figure 3:**
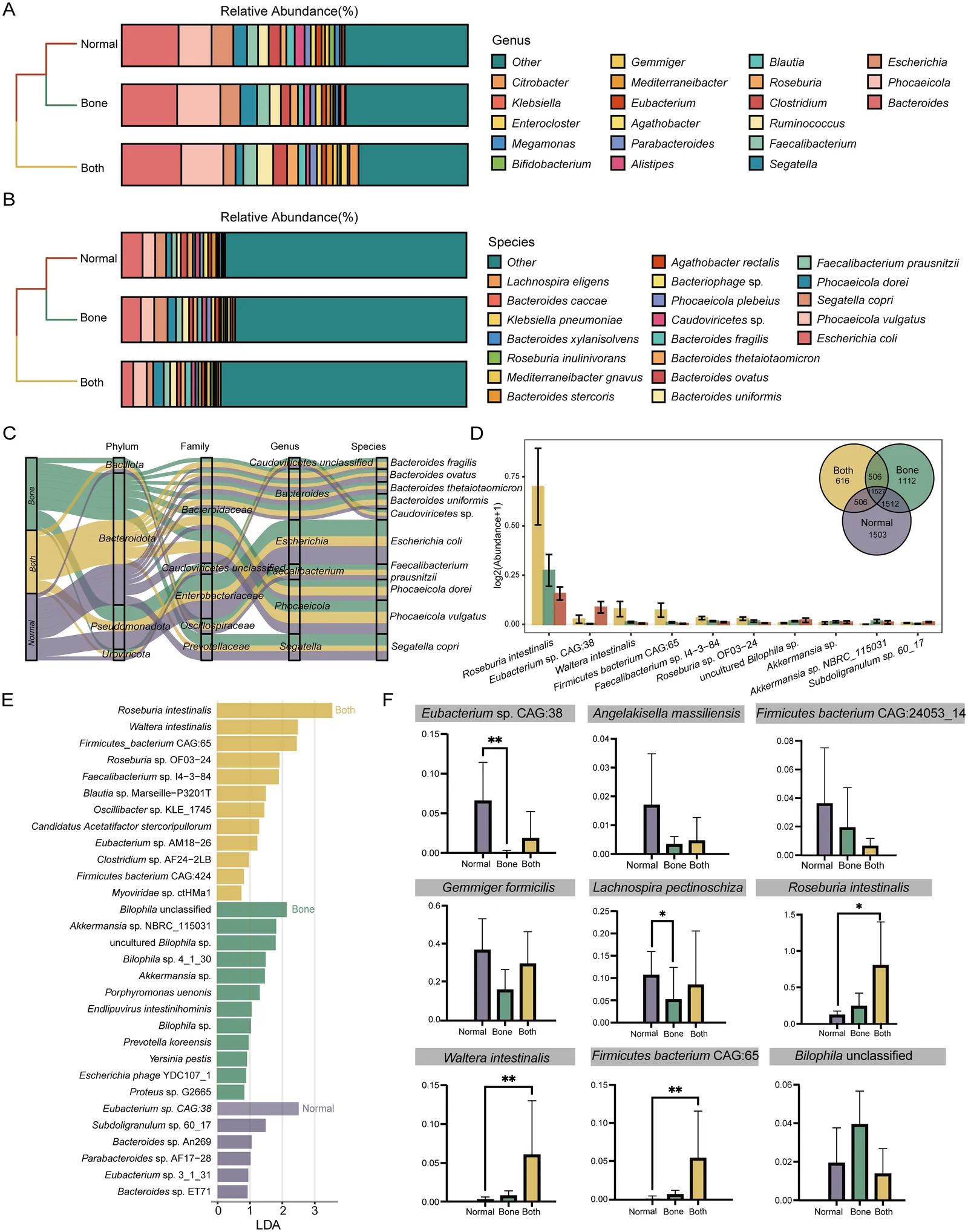
Microbiome composition across the Normal, Bone, and Both groups. **(a)** Genus-level relative abundance profiles in Normal, Bone, and Both. **(b)** Species-level relative abundance profiles in the same three groups. **(c)** Sankey diagram showing the taxonomic mapping of the top 10 species (by mean relative abundance across groups) from phylum to species. **(d)** Species showing nominal between-group differences in nonparametric testing, shown together with a Venn diagram summarizing species-level overlap. **(e)** LEfSe analysis (LDA > 2, *p* < 0.05) identifying group-discriminative species: Normal—*Eubacterium* sp. CAG:38; isolated low bone mass—*Bilophila* (unclassified); osteosarcopenia—*Roseburia intestinalis*, *Waltera intestinalis*, and *Firmicutes* bacterium CAG:65. **(f)** Relative abundances of nine consensus feature species retained after application of the predefined ≥2-method criterion and removal of duplicates.

Across the Normal, Bone, and Both groups, genus- and species-level compositions showed visible but non-discrete differences (**Figures 3a, b**). A multi-rank Sankey plot of the top 10 species traced their taxonomic assignments from phylum to species and illustrated the extensive sharing of core taxa among groups (**Figure 3c**). Nonparametric testing identified species with nominal between-group differences, with the top 10 ranked species displayed in **Figure 3d**. LEfSe analysis (LDA>2, p<0.05) identified group-associated taxa, including *Eubacterium* sp. CAG:38 in the Normal group, *Bilophila* (unclassified) in the Bone group, and *Roseburia intestinalis*, *Waltera intestinalis*, and *Firmicutes bacterium* CAG:65 in the Both group (**Figure 3e**). Species supported by both nonparametric testing and LEfSe were combined with the initial five-species consensus set, and duplicates were removed, yielding nine consensus feature species for this cross-sectional comparison (**Figure 3f**).

**Figure 4:**
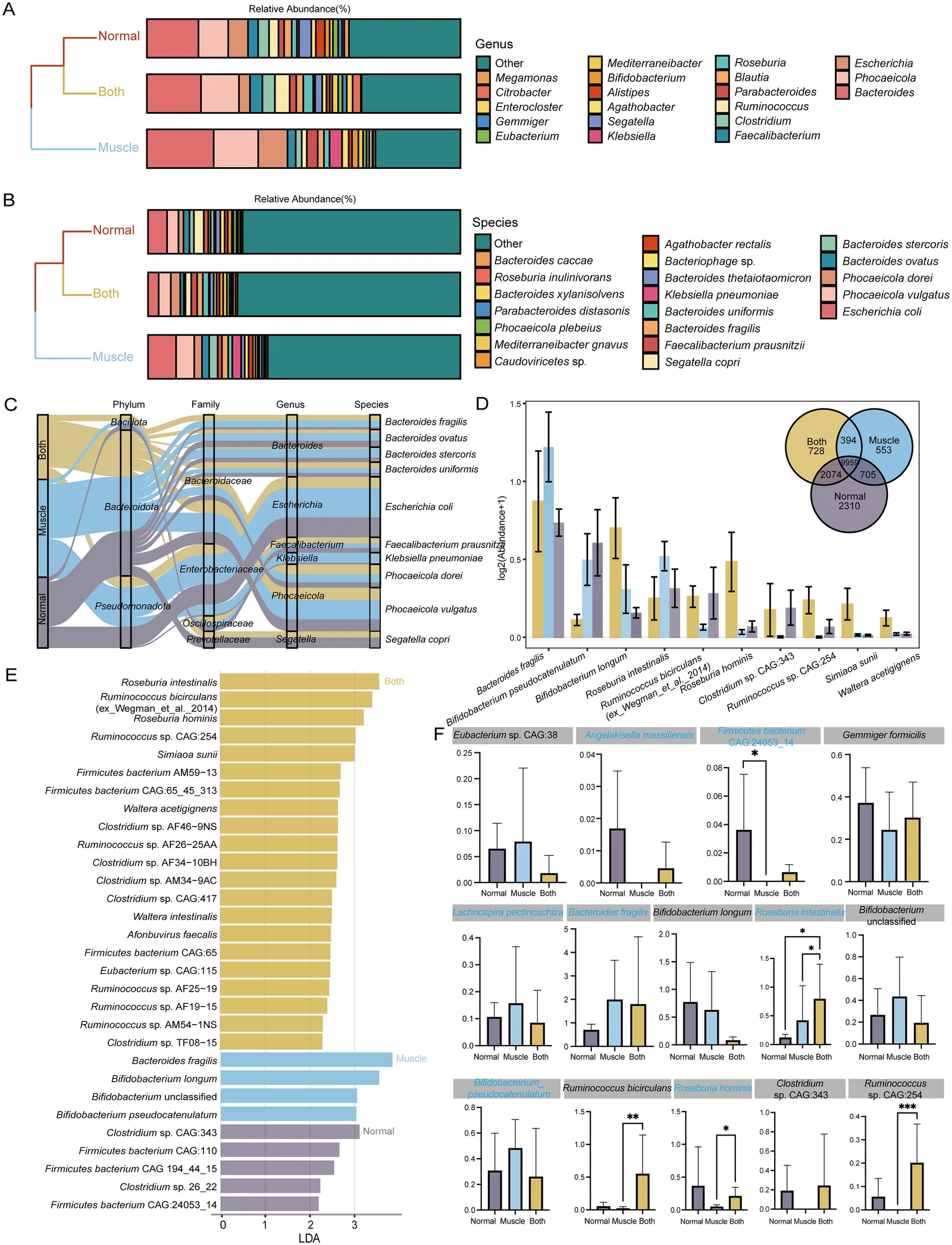
Microbiome composition across the Normal, Muscle, and Both groups. **(a)** Genus-level relative abundance profiles across the three groups. **(b)** Species-level relative abundance profiles. **(c)** Sankey diagram showing the top 10 species (ranked by mean relative abundance) mapped to higher taxonomic ranks. **(d)** Species showing nominal between-group differences in nonparametric testing, accompanied by a Venn diagram summarizing species overlap. **(e)** LEfSe results (LDA > 3, *p* < 0.05) identifying group-discriminative species: Normal—*Clostridium* sp. CAG:343; isolated sarcopenia—*Bacteroides fragilis*, *Bifidobacterium longum*, *Bifidobacterium* (unclassified), *Bifidobacterium pseudocatenulatum*; osteosarcopenia—*Roseburia intestinalis*, *Ruminococcus bicirculans*, *Roseburia hominis*, *Ruminococcus* sp. CAG:254, and *Simiaoa sunii*. **(f)** Relative abundances of 14 consensus feature species retained after application of the predefined ≥2-method criterion and removal of duplicates.

A parallel cross-sectional comparison among the Normal, Muscle, and Both groups similarly showed substantial taxonomic overlap, accompanied by visible group-associated differences at both the genus and species levels (**Figures 4a, b**). A multi-rank Sankey plot illustrated the taxonomic assignments of the top 10 species (**Figure 4c**). Nonparametric testing identified taxa with nominal between-group differences, with the top 10 ranked species displayed in **Figure 4d**. LEfSe analysis (LDA>3, p<0.05) identified group-associated taxa, including *Clostridium* sp. CAG:343 in the Normal group; *Bacteroides fragilis*, *Bifidobacterium longum*, *Bifidobacterium* (unclassified), and *Bifidobacterium pseudocatenulatum* in the Muscle group; and *Roseburia intestinalis*, *Ruminococcus bicirculans*, *Roseburia hominis*, *Ruminococcus* sp. CAG:254, and *Simiaoa sunii* in the Both group (**Figure 4e**). After application of the predefined ≥2-method consensus criterion and removal of duplicates, 14 consensus feature species were retained for this cross-sectional comparison (**Figure 4f**). These species were subsequently incorporated into the consolidated consensus feature set used for downstream descriptive and multi-omics analyses.

### 3.4 Consolidated consensus feature set and cross-group distribution

**Figure 5:**
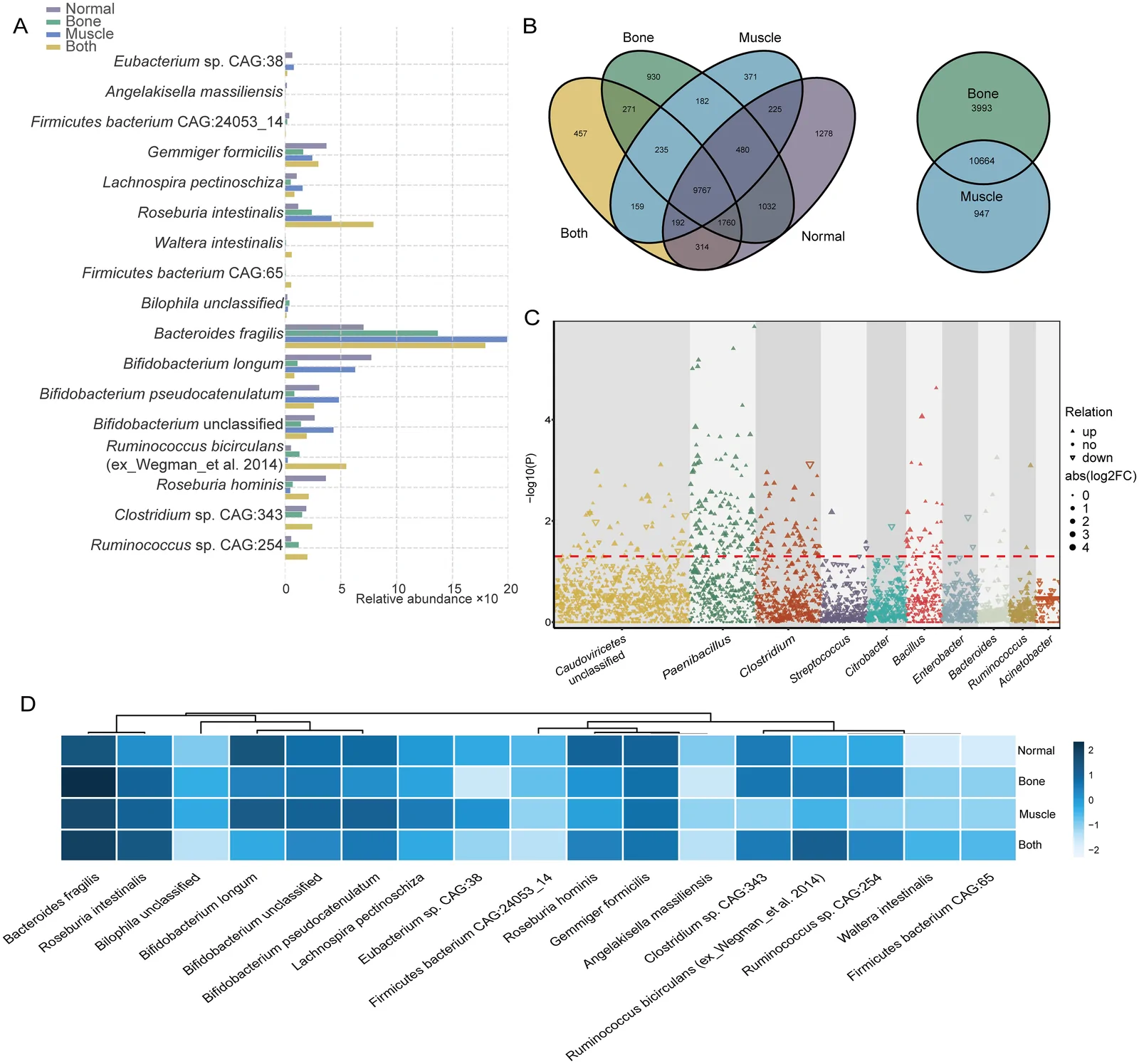
Integrated feature-species panel and higher-rank microbial differences across musculoskeletal phenotypes. **(a)** Mean relative abundances (×10) of the 17-species consensus feature set across the four groups—Normal, Bone, Muscle, and Both. These species met the predefined ≥2-method criterion across the corresponding comparison framework and were consolidated after removal of duplicates. **(b)** Venn diagrams showing species-level overlap. Left: four-group comparison illustrating shared and group-specific species; right: pairwise comparison between Bone and Muscle showing extensive overlap with distinct residual taxa. **(c)** Manhattan plot of nominal genus-level differential signals. Genera are ordered along the x-axis; the y-axis shows −log_10_ of the nominal *p* value. Point color indicates direction of change (up/down), and point size encodes |log_2_ fold change|. **(d)** Heatmap with hierarchical clustering of the 17 feature species across the four groups.

Building on the two cross-sectional phenotype comparisons, we consolidated non-redundant taxa meeting the predefined ≥2-method criterion, yielding a final 17-species consensus feature set for downstream descriptive and microbe–metabolite analyses (**Figure 5a**). Species-level Venn analysis showed a broadly shared microbial core together with group-specific taxa (**Figure 5b**). At the genus level, a Manhattan plot displayed the top 10 genera ranked by nominal statistical evidence (**Figure 5c**). Hierarchical clustering of the 17 species provided a descriptive overview of their relative abundance patterns across the four musculoskeletal phenotypes (**Figure 5d**).

### 3.5 Exploratory microbe–metabolite correlation network and predicted functional mapping

**Figure 6:**
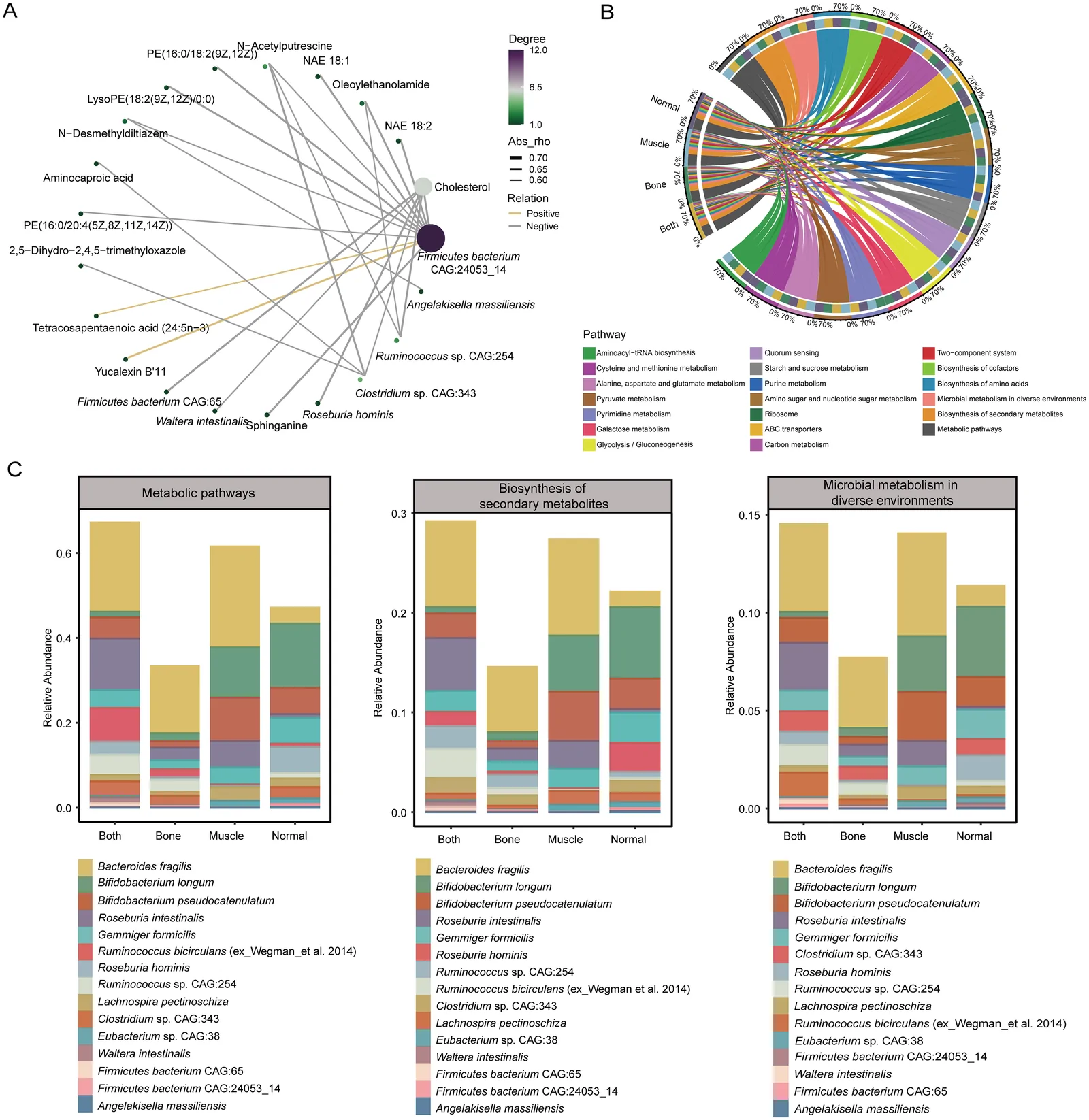
Exploratory microbe–metabolite correlation network and predicted functional mapping. **(a)** Exploratory network based on unadjusted Spearman correlations between the 17 consensus feature species and fecal metabolites in participants with paired multi-omics data (n=52). Edges meeting |ρ|≥0.7 and nominal *p* < 0.05 are displayed; no multiple-testing correction was applied. Node size and color indicate degree centrality, and edge color indicates positive or negative correlation. *Firmicutes bacterium* CAG:24053_14 showed multiple correlations with lipid-related features, including putatively annotated cholesterol and N-acylethanolamines. All named metabolites in this panel should be considered putative annotations rather than definitively identified compounds. **(b)** Chord diagram descriptively summarizing the distribution of predicted KEGG functional categories across the Normal, Bone, Muscle, and Both groups. Link width represents the relative contribution of each group to the corresponding predicted category. **(c)** Stacked bar charts showing the relative contributions of the 17 consensus feature species to three representative predicted KEGG categories: Metabolic pathways, Biosynthesis of secondary metabolites, and Microbial metabolism in diverse environments. These panels describe predicted microbial functional potential and do not directly measure metabolic flux, enzymatic activity, or causal pathway activity.

We next integrated the 17-species consensus feature set with the paired metabolomics data (n=52) to construct an exploratory microbe–metabolite correlation network. Using unadjusted Spearman correlations (|ρ|≥0.7, nominal p<0.05), the analysis identified several high-degree nodes (**Figure 6a**). All metabolite identities reported in this network should be considered putative annotations. *Firmicutes bacterium* CAG:24053_14 was correlated with several lipid-related features, including N-acylethanolamines (NAEs; such as oleoylethanolamide [OEA] and NAE 18:2), phosphatidylethanolamines [PE(16:0/18:2), PE(16:0/20:4), and LysoPE(18:2/0:0)], and cholesterol. Additional correlations involved sphinganine, aminocaproic acid, N-acetylputrescine, and tetracosapentaenoic acid (24:5n-3). Two features, N-desmethyldiltiazem and Yucalexin B’11, were putatively annotated as xenobiotic- or plant-derived compounds. Because these correlations were not adjusted for age or corrected for multiple testing, the network was used only to prioritize candidate associations and does not demonstrate direct microbial–metabolite interactions.

To provide functional context for these correlations, KEGG annotations were summarized across the four musculoskeletal phenotypes (**Figure 6b**). Frequently represented categories included Metabolic pathways, Biosynthesis of secondary metabolites, Microbial metabolism in diverse environments, Carbon metabolism, Biosynthesis of amino acids, Glycolysis/Gluconeogenesis, Nucleotide metabolism, Amino acid metabolism, and Transport and signaling systems. The stacked contribution analysis descriptively showed group-associated differences in the relative contributions of selected consensus feature species—including *Roseburia intestinalis*, *Ruminococcus* spp., *Bacteroides fragilis*, *Bifidobacterium* spp., and *Firmicutes bacterium* CAG:65 and CAG:24053_14—to three representative KEGG categories (**Figure 6c**). These analyses relate the candidate taxa to predicted microbial functional categories but do not directly measure metabolic flux, enzymatic activity, metabolite production, host responses, or causal pathway activity.

## 4 Discussion

In this cross-sectional study of fracture patients across four musculoskeletal phenotypes, the gut microbial communities did not separate into discrete group-specific configurations. Instead, the unadjusted analyses showed substantial taxonomic overlap accompanied by differences in the relative abundances of selected taxa. A multi-method analytical strategy yielded a 17-species consensus feature set. Taxonomic patterns were consistent with a candidate fiber/short-chain fatty acid (SCFA)-associated module, whereas exploratory microbe–metabolite correlations suggested a candidate lipid/sterol-associated module; predicted functional mapping provided descriptive context for these associations. These findings are hypothesis-generating and should not be interpreted as evidence of disease progression, causal pathways, direct microbial–metabolite interactions, or validated biomarkers.

### 4.1 The fiber/SCFA-associated module: heterogeneity among butyrate-producing taxa

Several group-discriminative taxa identified in our study belonged to well-characterized fiber-fermenting, butyrate-producing lineages, such as *Roseburia*, *Eubacterium*, *Lachnospira*, and *Gemmiger*. This finding aligns with a large body of evidence demonstrating that SCFAs influence bone and muscle metabolism through immunometabolic and epigenetic mechanisms (11, 15, 25, 26). Specifically, the microbial metabolite butyrate has been shown to be potently anabolic, constraining osteoclastogenesis and promoting osteoblast activity via a Treg-dependent Wnt10b signaling pathway (27, 28). In parallel, SCFAs support muscle energetics and protein turnover, with recent studies showing they can enhance muscle mass and function through the activation of mTOR signaling (12, 13).

Across the two cross-sectional phenotype comparisons, the butyrate-associated taxa did not show a uniform directional pattern. For example, *Eubacterium* sp. CAG:38 was enriched in the Normal group, whereas *Roseburia intestinalis* was enriched in the Both group. This heterogeneity may reflect differences in ecological context, species-specific functions, or other unmeasured host factors. However, fecal SCFA concentrations were not directly quantified, and the KEGG annotations reflect predicted microbial functional potential rather than measured metabolic activity. Therefore, the present data cannot establish either an overall reduction in SCFA production or a compensatory response among butyrate-producing taxa.

### 4.2 A candidate lipid/sterol-associated module in musculoskeletal phenotypes

Beyond the fiber/SCFA-associated module, the exploratory correlation network identified several lipid-related associations. *Firmicutes bacterium* CAG:24053_14 was correlated with putatively annotated cholesterol, N-acylethanolamines (NAEs), including OEA, and phosphatidylethanolamine-related features. Previous studies have described NAEs as bioactive lipids involved in the regulation of inflammation and lipid metabolism, partly through PPAR-α signaling. However, the present study did not directly measure the biological activity of these metabolites or establish that their variation was caused by the associated microbial taxa (29–31).

The gut microbiome has been implicated in host lipid homeostasis through bile acid and sterol transformations (9, 10). Furthermore, large-scale population studies have reported associations between microbial composition and circulating or fecal lipidome profiles, including cholesterol (22, 32–35). In the present study, KEGG annotations provided descriptive functional context for the observed correlations but did not directly corroborate microbial regulation of the lipid metabolites. Accordingly, the observed pattern is described as a candidate lipid/sterol-associated module rather than an established functional axis. The findings do not demonstrate that the identified taxa produced or regulated the putatively annotated metabolites, nor do they establish that this module contributes causally to osteosarcopenia. Targeted metabolomics, authentic-standard confirmation, and experimental studies are required to evaluate these candidate relationships.

### 4.3 Clinical context

Age differed significantly across the four groups, with the Bone group having the highest mean age. Aging is a known driver of microbiome remodeling, characterized by the loss of SCFA producers, altered bile acid metabolism, and low-grade inflammation (16, 36, 37). Because age was not incorporated as a covariate in the differential-abundance or correlation analyses, the observed microbial and metabolite differences—particularly those involving the Bone group—cannot be distinguished from age-related variation. We therefore regard these findings as unadjusted exploratory associations.

The predicted KEGG categories included lipid handling and central carbon–amino acid metabolism; however, these annotations represent microbial functional potential and do not establish pathway activity or a biological mechanism underlying musculoskeletal dysfunction. These exploratory patterns may nevertheless help define questions for future translational studies. Preclinical and clinical literature has suggested that probiotic interventions may influence bone metabolism, although the available evidence remains heterogeneous (38–40). These external findings provide a rationale for future interventional research but do not establish the clinical utility of the candidate modules identified in the present study. The current analysis did not evaluate diagnostic accuracy or treatment response and therefore does not support the use of microbiome–metabolome profiles to replace or complement DXA in clinical practice.

### 4.4 Strengths, limitations, and future directions

Methodologically, the study combined paired shotgun metagenomics and untargeted metabolomics with preprocedural fecal sampling and a multi-method consensus feature-selection strategy. Several limitations should nevertheless be considered. First, the cross-sectional design precludes temporal or causal inference. Second, the modest overall sample size and the small phenotype subgroups, particularly the 11–16 participants per group with paired multi-omics data, limit statistical power and the stability of feature selection and network estimation. The 17-species consensus feature set has not been validated in an independent cohort and should not be regarded as a validated microbial signature. Third, the analyses were not adjusted for age, leaving residual confounding particularly relevant to comparisons involving the older Bone group. Fourth, calf circumference was used as a surrogate for muscle mass because DXA- or BIA-derived appendicular muscle mass was unavailable, potentially resulting in misclassification of the Muscle and Both phenotypes. Additional analytical limitations include the use of fecal rather than circulating metabolites, the absence of authentic-standard confirmation for the key metabolite annotations, and the use of nominal p values without multiple-testing correction in the exploratory correlation network. Moreover, KEGG annotation reflects predicted microbial functional potential rather than directly measured metabolic flux or enzymatic activity. These limitations reinforce the hypothesis-generating nature of the identified microbe–metabolite associations.

Future studies should recruit larger, age-balanced cohorts and use DXA- or BIA-derived appendicular muscle mass to confirm sarcopenia phenotypes. Longitudinal sampling and independent external validation will be necessary to determine the reproducibility and temporal relevance of the 17-species consensus feature set. Targeted assays using authentic standards should quantify fecal and circulating SCFAs, OEA, cholesterol, and related lipid mediators, while confirmatory multi-omics analyses should incorporate prespecified covariate adjustment and multiple-testing control. If the candidate fiber/SCFA- and lipid/sterol-associated modules are replicated, subsequent intervention studies could evaluate dietary fiber, prebiotics, probiotics, or lipid-signaling strategies using bone density, appendicular muscle mass, muscle strength, physical performance, and fracture-related outcomes.

## 5 Conclusions

In this exploratory cross-sectional study of fracture patients, the four musculoskeletal phenotypes shared a broadly overlapping gut microbial community but showed unadjusted differences in selected microbial taxa and putatively annotated fecal metabolites. A multi-method strategy yielded a 17-species consensus feature set. Taxonomic patterns were consistent with a candidate fiber/SCFA-associated module, whereas exploratory microbe–metabolite correlations suggested a candidate lipid/sterol-associated module. These associations do not establish direct microbial–metabolite interactions, causal pathways, validated biomarkers, or therapeutic targets. Their interpretation is limited by the modest sample size, age imbalance, operational assessment of sarcopenia using calf circumference, absence of authentic-standard metabolite confirmation, lack of multiple-testing correction in the correlation network, and absence of external validation. Larger, age-balanced cohorts using DXA- or BIA-based muscle phenotyping, targeted metabolomics, prespecified covariate adjustment, and independent validation are required to determine the reproducibility and potential clinical relevance of these findings.

## Data Availability

The datasets presented in this study can be found in online repositories. The names of the repository/repositories and accession number(s) can be found below: https://ngdc.cncb.ac.cn/bioproject/browse/PRJCA041726, CRA027769.
